# An electrochemical sensor for the detection of arsenic using nanocomposite-modified electrode

**DOI:** 10.1038/s41598-023-36103-6

**Published:** 2023-05-31

**Authors:** Sara Hamid Kargari, Fatemeh Ahour, Mehdi Mahmoudian

**Affiliations:** 1 Department of Nanotechnology, Faculty of Chemistry, Urmia University, Urmia, Iran; 2 Institute of Nanotechnology, Urmia University, Urmia, Iran

**Keywords:** Chemistry, Materials science, Nanoscience and technology

## Abstract

The aim of this research is to develop an electrochemical sensor based on a conducting polymer, polyaniline, and a cationic polymer, poly(diallyldimethylammonium chloride), reinforced with graphene oxide nanosheets functionalized with acrylic acid. The two-dimensional nature of acrylic acid functionalized graphene oxide nanosheets and clusters made of conductive polymers and acrylic acid functionalized graphene oxide nanosheets were confirmed by microscopic tests. The prepared nanocomposite was deposited on the glassy carbon electrode in order to prepare an electrochemical sensor for the detection of arsenic by cyclic voltammetry and differential pulse voltammetry methods. It should be mentioned that the presence of acrylic acid functionalized graphene oxide nanosheets increases the surface area due to the nano size effect and better dispersion of this nanomaterial, poly(diallyldimethylammonium chloride), increases the adsorption capacity of the analyte due to electrostatic interaction between the negatively charged analyte and positively charged surface, and polyanilin increases the charge transfer rate due to the good conductivity. The results show that the prepared electrode has a sensitivity equal to 1.79 A/M with 0.12 μM as the detection limit. The proposed sensor could be used for the determination of total inorganic arsenic by first oxidative pretreatment for conversion of As(III) to As(V).

## Introduction

Among the types of heavy and harmful metals for humans and animals, arsenic is considered one of the most dangerous metals for the environment, which threatens the lives of millions of people^1^. Between the different chemical species of arsenic (arsenite, arsenate, and organic derivatives of this metal), its mineral states are more toxic in nature due to higher ionic mobility than organic species. According to the rules of the World Health Organization, it is necessary to remove arsenic from natural and drinking water until the concentration below 10 ppb (0.14 μM) is reached^2^.

The known analytical methods for determining arsenic in water are inductively coupled plasma-mass spectrometry, absorption, and emission atomic spectroscopy methods, atomic fluorescence spectroscopy, high-performance liquid chromatography, and ultra-functional chromatographic methods, which require complex and expensive instruments, so the use of these methods has limitations and cannot be suitable for field analysis^3–8^.

Electrochemical analytical methods have shown in recent years that they are a promising approach and can replace classical methods for the quantitative and qualitative detection of arsenic. Electrochemical diagnostic systems have advantages such as simplicity in instrumentation, high sensitivity, selectivity, and convenience. Moreover, these methods have the ability to be miniaturized, which allows them to be used at different times and places^9^. One of the parameters affecting the electrode processes is the electrode surface characteristics, therefore, by changing the electrode surface by fixing a suitable reagent, analytical goals such as sensitivity and selectivity of chemical reactions can be increased. Considerable research has been done on the development of electrochemical sensors to detect arsenic mineral species in the environment. In this regard, various methods have been proposed to modify electrodes and achieve the desired improvement in results^10–12^.

Designing and manufacturing electrodes modified with nanomaterials has attracted attention, and several scientific reports have been published in this field in recent years^13–16^. Nanoparticles have unique physical, chemical, electronic, and optical properties, and introducing them into the electrode structure can induce these features in electrochemical sensors. Modification of electrodes with nanomaterials, for electrochemical purposes and detection of heavy metals, improves the sensitivity of the sensor due to the high surface-to-volume ratio and better conductivity^17^.

Modified electrodes with ruthenium nanoparticles decorated on glassy carbon electrodes (GCE)^18^, thiol functionalized carbon nanotubes^19^, graphene–lead oxide composite^20^, nanocauliflower structured fluorine doped cadmium oxide (CdO) thin film^21^, bimetallic gold and copper nanoparticles^22^, platinum nanoparticle modified boron doped diamond microelectrodes^11^, manganese-coated gold microwire^23^, double-walled carbon nanotubes and graphene hybrid thin film^24^, Magnetite-Decorated Gold Nanoparticles (Fe_3_O_4_-Au)^25^, gold nanoparticles (AuNPs) functionalized single polypyrrole nanowires (PpyNW)^26^, gold nanoparticles^27^, and aptamer^1^, have been used for arsenic detection and reported in review articles. Two-dimensional graphene oxide nanosheets (GO) with a wide cross-sectional area and high mechanical and thermal resistance have unique electronic properties, which makes them a suitable option for modifying the electrode surface^13^. Mineral arsenic exists as oxoacid with a negative charge, thus positively charged modifier could improve its adsorption and detection. In this regard, poly (diallyl dimethyl ammonium chloride) (PDDA) has a quaternary ammonium salt structure, and the positive charge of quaternary ammonium salt on this polymer has given it hydrophilic properties. So that this polymer is soluble in water and can be used in the preparation of polyelectrolytes^28^. This polymer has been announced as the first polymer approved by the US Food and Drug Administration and is used in various industries, including the manufacture of electrochemical sensors and solar cells. The electrical conductivity of PDDA is not good enough, which may limit its development and application to some extent. Therefore, composing this polymer with conductive materials can improve its properties and give it interesting features^29–33^.

Among conductive polymers, polyaniline (PA) has attracted special attention due to its features such as easy synthesis, low price, wide application, and high polymerization efficiency. The electrical, electrochemical, and optical properties of PA have made it an attractive product for use in electronic industries, and anti-static and anti-corrosion coatings^34^. However, PA has disadvantages like limited processability and low mechanical properties^34–37^. Therefore, blending polyaniline with PDDA could improve the processability of PA and gain desired properties.

Polymeric films are deposited on the surface of the electrode both from the solution containing monomer and polymer. Deposition of the polymeric film on the electrode is done through immersion or rotation of the electrode in the solution. Moreover, a variety of grafting techniques or electrochemical deposition are also among the conventional methods for forming a polymeric layer on the electrode. Also, thermal, electrochemical, plasma, or photochemical deposition can be used to prepare polymeric films from monomer solutions. The mechanical and electrical properties of these polymers are directly related to the doped species in their structure^34–39^. So far, some polymer composites have been used as electrode modifiers for sensor applications^40–48^. It should be noted that polymers such as polyvinylidene and (E)-N′-(2-nitrobenzylidene)-benzenesulfonhydrazide were deposited on the electrode surface and were used for the determination of As concentration with interesting results^49,50^.

**Figure 1:**
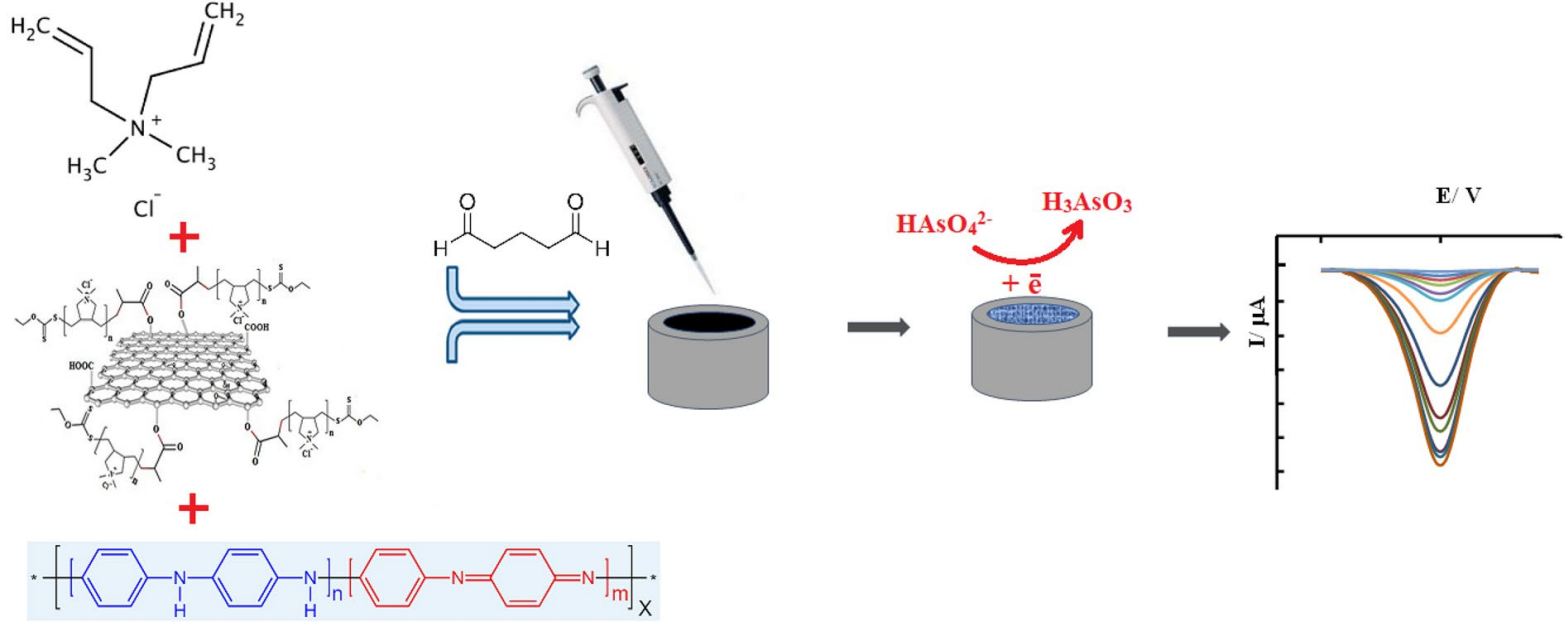
Schematic presentation of prepared sensor.

In the present work, the copolymer of PA and PDDA doped with acrylic acid-functionalized graphene oxide (AAGO), was used to prepare an arsenic sensor (Fig. 1).

The proposed sensor has high conductivity due to the presence of a conductive polymer. In addition, the positively charged PDDA increases the adsorption of negatively charged arsenate species as reported previously for similar polymers^51^. Also, the application of AAGO due to its nanometer size increases the active surface area and improves the resulting signal. The functionalization of graphene oxide also makes its better spread in the polymer substrate. In simpler terms, it can be said that these components have a synergistic effect and lead to the development of a sensor with excellent characteristics in terms of selectivity and sensitivity.

## Experimental

### Materials and instruments

Deionized water was used to prepare the solutions. All chemicals consisting Graphite, acrylic acid, aniline, 2-bromopropinyl bromide (BPB, 97%), potassium ethyl xanthate, potassium permanganate, ammonium persulfate, diallyl dimethylammonium chloride, carbon disulphide and ethanol (synthetic grade), pyridine, *N-*methylpyrrolidone (NMP), *N,N*-dimethylformamide (DMF), acetone, chloroform, dichloromethane and diethyl ether, N,N'-Methylenebisacrylamide (MBA), azobisisobutyronitrile (AIBN), phosphoric acid, sulfuric acid, ammonium nitrate, hydrogen peroxide (30%), hydrochloric acid and potassium hydroxide) were purchased from Sigma-Aldrich. Sodium dihydrogen arsenate (NaH_2_AsO_4_), arsenic (III) trioxide (As_2_O_3_), nitrate salt of metals cations (Ag^+^, Cu^2+^, Co^2+^, Cd^2+^, Pb^2+^, Fe^2+^), tyrozin, acetaminophene, isoniazide and ascorbic acid and other analyts of anlytical grade were purchased from Sigma-Aldrich and used without further purification. Stock solutions of analytes (0.1 M) were prepared from the purchased chemicals as per the experimental requirements. Analytical solutions were prepared using deionized water. IR spectra were recorded by a Nicolet FT-IR NEXUS 670 spectrometer (Thermo Scientific, USA) and used to identify the presence of functional groups in the synthesized compounds. TESCAN MIRA III scanning electron microscope was used to record FE-SEM, EDAX and mapping images. pH was measured using a digital pH meter (HANNA 212). An ultrasonic bath (KODO model JAC1002) was used to clean the GCE surface and prepare homogeneous suspensions of modifiers.

### Preparation of GO

For the synthesis of graphene oxide, the modified Hummer method was used. In this method, initially, graphite powder (5 g) was dispersed in a mixture of H_3_PO_4_ (12 ml) and H_2_SO_4_ (100 ml) for 60 min. In the next step, ammonium nitrate (5 g) was added to the mixture. In continue, KMnO_4_ (3 g) was added, and stirring was continued in a ice bath at 5 °C for 2 h. Then the temperature was raised up to 98 °C, so that the process of oxidation and exfoliation can be done in the best way. Graphene oxide was stirred under these conditions for 1 h and then distilled water was slowly added to the solution along with 20 ml of hydrogen peroxide (30%) to complete the oxidation process. Finally, the resulting sediment was washed several times with distilled water to neutralize its pH. The prepared sediment was separated by centrifugation at a speed of 5000 rpm and dried at 60 °C.

### Functionalization of GO with acrilic acid

For the synthesis of graphene oxide, the modified Hummer method was used as described in SI. To create the acrylic acid functionality on the surface of GO, 2-bromopropionyl bromide (BPB) was used as a linker to anchor the xanthate agent on the surface. For this purpose, GO (250 mg) was dispersed in pyridine (40 ml) and deoxygenated with a stream of nitrogen gas, and then 3 ml of BPB was added drop by drop to the reaction. The mixture was stirred for 3 h at a temperature of 0 °C and 48 h at a temperature of 25 °C until the reaction was complete and finally, the sediment was washed three times with chloroform to separate unreacted BPB. The product BPB modified GO (GO-BPB) was dried under a vacuum at 50 °C.

Potassium ethyl xanthate was used to create xanthate groups, which is the initiator of reversible addition-fragmentation chain transfer polymerization (RAFT) method. This reagent was synthesized and used according to our previous report^52^.

GO-BPB (100 mg) and potassium ethyl xanthate (0.5 g) were mixed together and poured into a round-bottom flask and degassed after sealing. The mixture of dichromethane (8 ml) and pyridine (3 ml) was degassed in another vessel and added to the container containing the ingredients with a syringe. This mixture was stirred at room temperature for 48 h and then diluted with dichloromethane (50 ml). The obtained sediment after centrifugation was dried at 50 °C under a vacuum.

Polymerization of acrylic acid on the surface of graphene oxide was done by the RAFT method. In this reaction, xanthate fixed on graphene oxide acts as an initiator. Graphene oxide with xanthate groups (50 mg) along with 3 mg azobisisobutyronitrile (AIBN) was dissolved in 2 ml dimethylformamide (DMF) and degassed. Acrylic acid (2 ml) was degassed in another container and injected into the container containing modified graphene oxide. The reaction was continued at 90 °C for 48 h and then the resulting black precipitate was washed several times with acetone and distilled water and dried at 50 °C under vacuum.

### Synthesis of polyaniline

To prepare polyaniline, two containers were needed. In a sealed container, aniline (0.5 ml) was mixed with 1 M hydrochloric acid solution (50 ml). In the second vessel, an oxidizing solution was prepared by combining ammonium persulfate (1.55 g) with 1 M hydrochloric acid (50 ml). The solutions of both containers were placed in an ice water bath until the temperature reach to 0 °C. The first container was degassed using nitrogen, and then the solution prepared in the second container was added drop by drop to the first one over a period of 1 h and the resulting precipitate was filtered, washed with a mixture of water and ethanol and finally stirred in an ammonia solution (1 M) for 48 h. The prepared green color sediment confirms the formation of polyaniline doped with ammonia, the resulting sediment was centrifuged, separated and dried at 40 °C.

### Synthesis of AAGO doped PDDA-PA nanocomposite (AAGO-PDDA-PA)

The method used to prepare the nanocomposite applied on the electrode surface has been reported in previous studies^52^. Diallyl dimethylammonium chloride (0.5 g) was poured into a solution of AAGO containing 1 mg/ml. Then, ammonium persulfate was added to the above solution 5 wt%. After the complete dissolution of the initiator in this solution, N,N′-methylenebisacrylamide (MBA) as cross-linking agent was added to the solution 30 wt% of the weight of the monomer. This solution was thoroughly stirred until a clear solution was prepared. In the next step, 0.003 g of polyaniline was added to the above solution. An ultrasonic probe was used to disperse this solution completely. Finally, this mixture was used to modify the electrode surface.

### Preparation of nanocomposite-modified electrode

In this research, the casting method was used to modify the electrode surface. For this purpose, 0.5 μl of the nanocomposite solution prepared in the previous step was dropped on the surface of the electrode using a sampler and dried at 40 °C for 24 h. The prepared electrode was used in quantitative arsenic detection experiments.

### Preparation of real samples

Commercial rice powder was purchased from the local market in Tabriz with the brand Golha and treated according to the published treatment process^53^.

For this purpose, 1 g of this fine powder of rice samples was dissolved in 10 ml of methanol–water mixture (1:1) containing 1% HNO_3_. The mixture was placed in an ultrasonic bath for 30 min and then centrifuged at 6000 rpm for 5 min. Then 1 ml of the sample solution supernatant was transferred to a 10 ml volumetric flask and diluted with acetate (dilution factor 1 g:100 ml). Tap water samples was prepared by adding a suitable amount of background electrolyte (1:100) without additional treatment. 5 ml of these real samples were used for analysis by the standard addition method.

### Electrochemical measurements

The AUTOLAB PGSTAT 30 device was used to perform all electrochemical measurements, including a conventional three-electrode: Ag/AgCl as a reference, modified GCE as working, and platinum wire as counter electrodes. Tow analytes were used in this research: 1) arsenic (III) trioxide (As_2_O_3_) and 2) Sodium dihydrogen arsenate (NaH_2_AsO_4_). As_2_O_3_ was dissolved in a minimum volume of concentrated sodium hydroxide solution and then it was brought up to volume in a volumetric flask. Before conducting the experiment, the pH of the above solution was acidified using hydrochloric acid (pH = 9 in this case), and the tests related to the analyte detection were performed by cyclic voltammetry (CV) method in the potential range from − 1 to + 2 V. NaH_2_AsO_4_ was dissolved in deionized water and CV or differential pulse voltammetry (DPV) electrochemical experiments were done in the potential range − 1 to 0 V with a pulse amplitude of 50 mV after adjusting solution pH in the desired value (4 in this case). The measurement of arsenate includes the initial accumulation of As(V) by immersing the modified electrode in the stirred solution of the analyte for 5 min, which is followed by the voltammetric measurement of the accumulated species.

The Fe(CN)_6_^3–^/Fe(CN)_6_^4–^ was used as a redox probe to evaluate the surface changes and modifier immobilization at the electrode surface. This evaluation was done by CV experiments in the potential range from − 1 to 1 V.

In order to investigate the electrochemical behavior of modified electrodes in arsenic analysis, a certain amount of As(III) or As(V) standard solution was added to the electrochemical cell containing 5 ml of background electrolyte with optimal pH and after placing the three-electrode system, the corresponding electrochemical response recorded after 300 s preconcentration.

## Results and discussion

In this article, it has been tried to use a new blended nanocomposite consisting of poly(diallyl dimethylammonium chloride), polyaniline, and graphene oxide modified with polyacrylic acid to stabilize on the electrode surface. This electrode is used to detect and measure arsenic oxoanions.

Poly(diallyl dimethylammonium chloride), having positive charge units in its repeating units, is very effective in interacting with anions and adsorbing them. Polyaniline was used as a conductive polymer to increase the electrical conductivity of the stibilized layer, and modified graphene oxide nanostructures by increasing the specific surface area and having chelating groups play an active role in adsorbing compounds on the electrode surface and increasing selectivity and sensitivity.

### Characterization of nanocomposite

**Figure 2:**
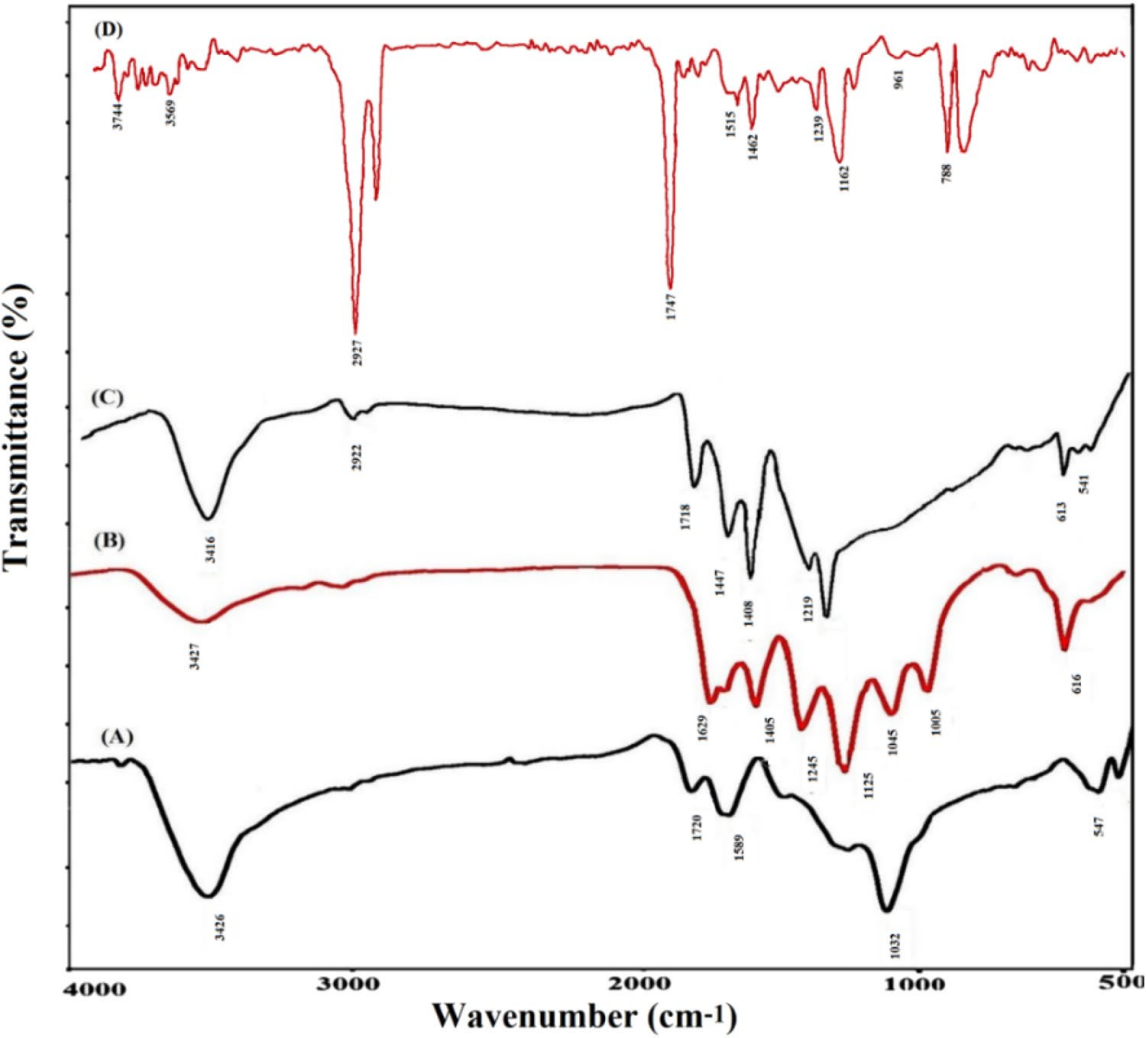
FT-IR spectra of (**A**) GO, (**B**) xanthate modified GO, (**C**) polyacrylic acid modified GO, and (**D**) AAGO-PDDA-PA nanocomposite.

Fourier-transform infrared spectroscopy (FT-IR) has played a substantial role in the tracking of functional group addition, elimination, and conversions in synthetic reactions. FT-IR spectroscopic study was utilized to confirm the sequential synthesis of modified graphene oxide and nanocomposite. The spectrum of graphene oxide at different stages of modification was recorded with this analysis and the obtained spectra are shown in Fig. 2. The characteristic peaks of GO appeared at the wavelengths of 3426, 1720, 1600, 1220, and 1060 cm^–1^, which respectively correspond to the stretching vibrations of the hydroxyl group, the stretching vibrations of the carbonyl group, the double bond vibrations of the graphite structure, and the stretching vibrations of the C–OH bond. In the spectrum related to GO modified with xanthate, a sharp decrease in the intensity of stretching vibrations of the hydroxyl group at 3400 cm^–1^ was observed, which indicates their reaction with xanthate groups. In addition, new peaks have appeared at 1125 and 1045 cm^-1^, which are related to CS_2_ groups and asymmetric stretching of the C–O–C bond, respectively.

In the spectrum of graphene oxide modified with polyacrylic acid, a peak at 1718 cm^–1^ appeared again, which is related to carboxylic acid groups. The peak at 2922 cm^–1^ is related to the aliphatic C–H bond. The appearance of these peaks confirms the successful modification of the graphene oxide surface with polyacrylic acid. In the spectrum related to the modified electrode with nanocomposite containing PDDA, PA, and AAGO nanoparticles, the presence of these substances can be proved according to the existing peaks. The peaks appearing at 2927, 1162, and 961 cm^–1^ correspond to the stretching vibrations of –CH_2_, C–N, and quaternary ammonium bonds in the poly(diallyldimethylammonium chloride), respectively.

The characteristic peaks of polyaniline have been observed at 3569, 3055, (1468 and 1515), 1239, and 788 cm^–1^ and are related to the stretching vibrations of amine groups, aromatic C-H, (quinonoid, benzenoid), C=N bond, and out-of-plane vibrations of C-H bonds, respectively. The presence of modified graphene oxide with polyacrylic acid can be proved by the presence of a sharp peak at 1747 cm^–1^.

**Figure 3:**
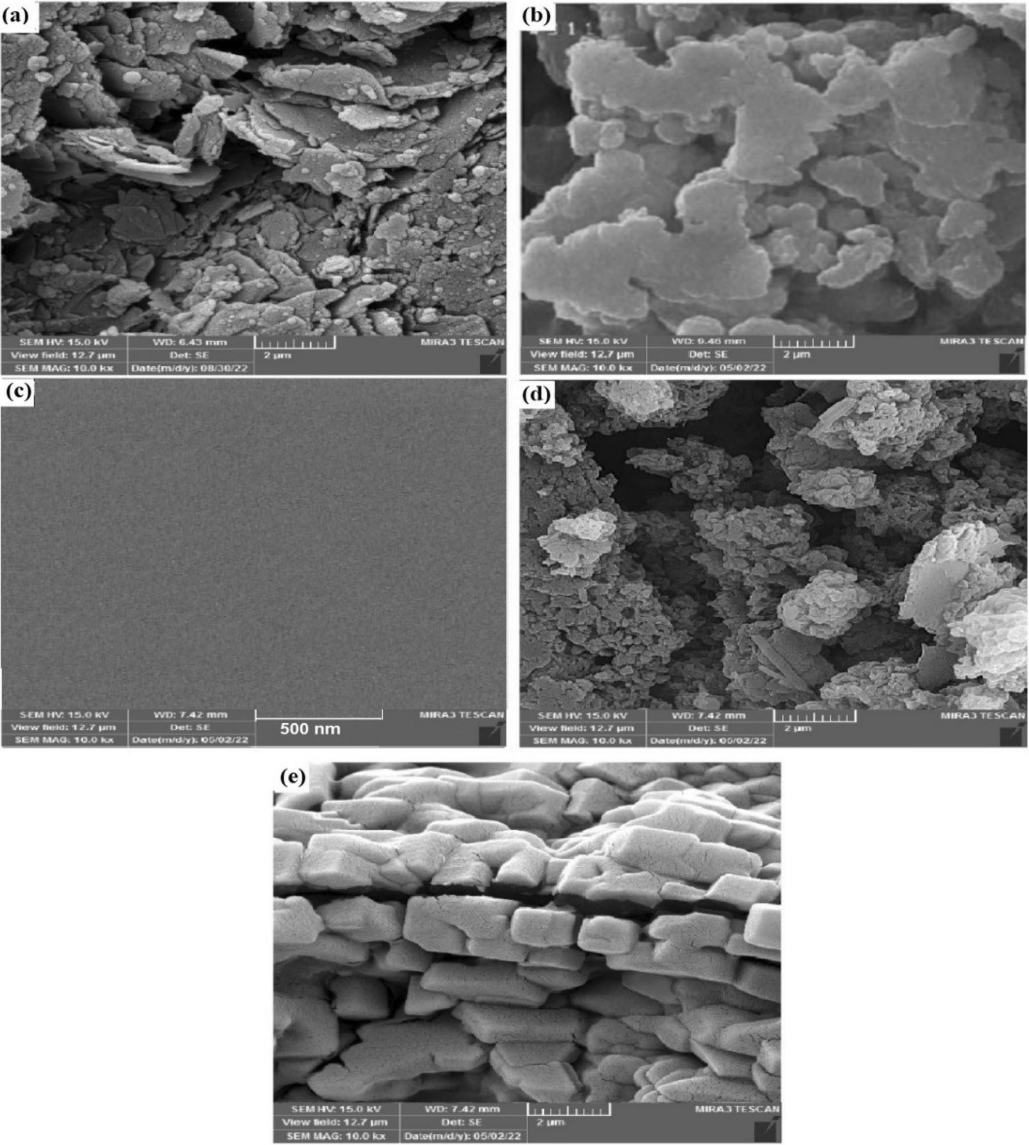
FESEM image of (**a**) GO, (**b**) AAGO, (**c**) bare GCE, (**d**) AAGO modified GCE, and (**e**) AAGO-PDDA-PA modified GCE.

The field emission scanning electron microscope (FESEM) technique was used to investigate the morphology of graphene oxide, modified graphene oxide with polyacrylic acid, as well as uncoated and coated electrodes. The resulting images are shown in Fig. 3.

The layered structure of graphene oxide can be recognized clearly in the image (a) and these layers have a smooth surface. In the next image (b), the covering of graphene oxide layers with polyacrylic acid can be seen and an uneven surface has been obtained.

To check the changes in the electrode surface, the images of the uncoated electrode (c), the electrode which was layered with modified graphene oxide (d), and the electrode coated with AAGO-PDDA-PA nanocomposite (e) were compared. The surface of the uncoated electrode was completely smooth, while structures similar to image (b) have been observed on the surface of the modified electrode, which was a confirmation of the successful stabilization of modified graphene oxide. The image obtained for the AAGO-PDDA-PA nanocomposite coated electrode showed regular three-dimensional structures that may be related to crystals formed from polyaniline or polydiallyldimethylammonium chloride salts.

**Figure 4:**
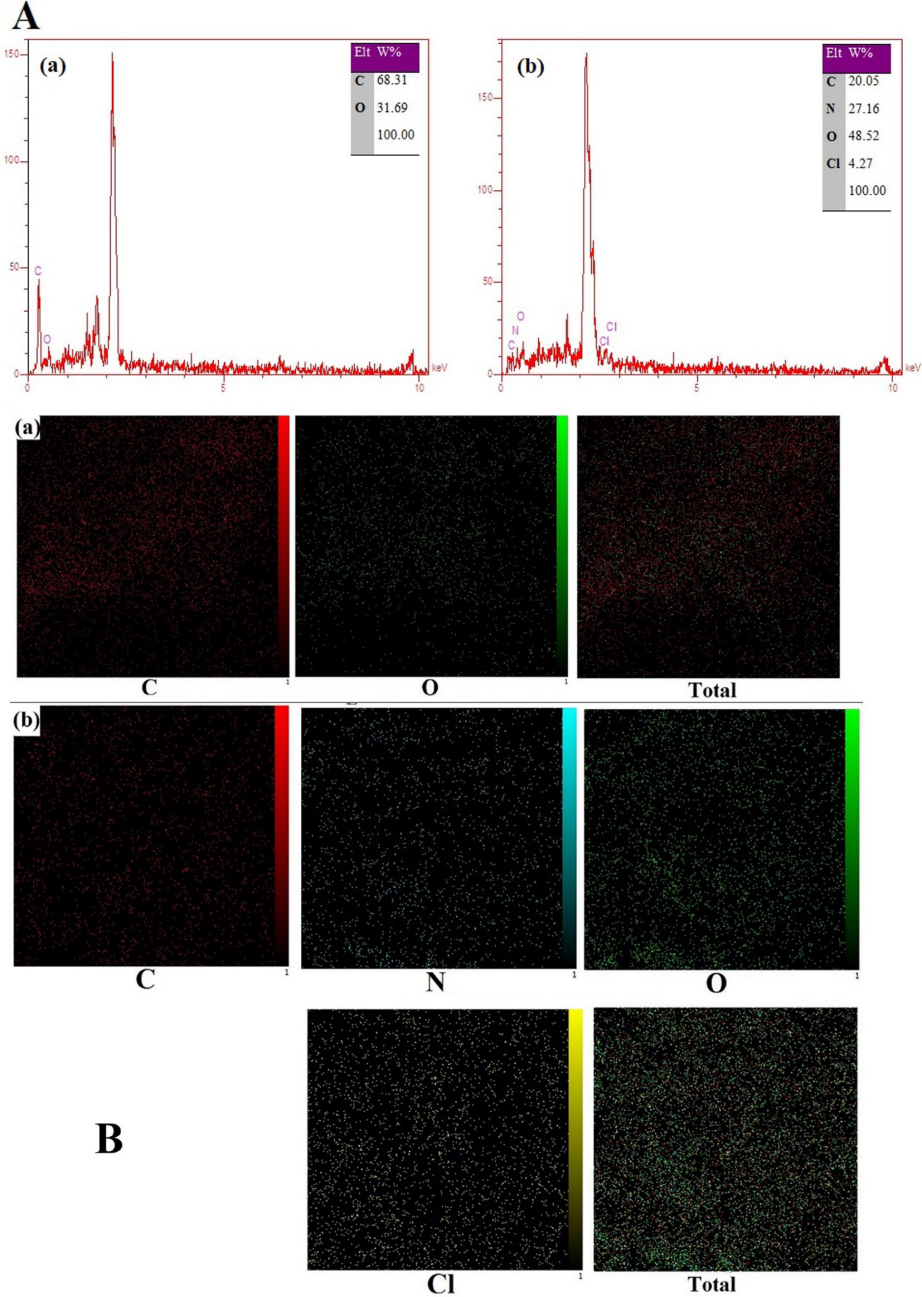
EDX spectra (**A**) and Elemental mapping (**B**) of the (**a**) AAGO and (**b**) AAGO-PDDA-PA nanocomposite.

To confirm the modification of the electrode surface, samples with stabilized modified graphene oxide and nanocomposite were analyzed by energy-dispersive X-ray spectroscopy (EDAX) elemental analysis. The results are shown in Fig. 4.

In the electrode modified with graphene oxide, oxygen, and carbon elements have been detected, while in the electrode covered with nanocomposite, there were other elements such as nitrogen and chlorine, which were present in the structure of the polyaniline and poly(diallyldimethyl ammonium chloride) polymers and showed the formation of a polymeric layer on the surface of the electrode. To check the dispersion of these elements on the surface of the electrode, mapping analysis was also performed on the samples and the results are shown in Fig. 4.

In the AAGO modified electrode, the dispersion of oxygen elements and in the nanocomposite sample, the dispersion of nitrogen and chlorine elements confirm the formation of uniform layers on the electrode surface.

### Preliminary experiments

The electrocatalytic performance of electrodes prepared with graphene oxide and different ratios of polymers were investigated by performing cyclic voltammetry in 5 mM $Fe(CN{)}_{6}^{3-}/Fe(CN{)}_{6}^{4-}$ (ferri-ferro) solution containing 0.5 M sodium chloride in the potential range from -1 to + 1 V with a scan rate of 50 mV/s (Fig. S1a).

The obtained results approve the successful immobilization of modifiers. As can be seen, with the addition of the polymers, the signal related to the ferri-ferro pair is also intensified, which may be related to the positive charge of the conductive polymers and the presence of electrostatic interaction between the negatively charged probe and electrode surface. In addition, at these electrodes, the current related to the electric double layer also increases due to the increase of surface charge. Also, the distance between anodic and cathodic peaks decreases may be due to the increased conductivity related to the conductive nature of polymers. Similarly, the presence of AAGO beside the polymers results in a current increase related to the increased surface area due to its nanostructure.

Also, to determine the electrochemically active surface area of the nanocomposite modified electrode, chronocoulometry in 1 mM K_3_[Fe(CN)_6_] containing 0.5 M NaCl solution was used, and based on the Anson equation ($Q=\frac{2nFAC{D}^{1/2}{t}^{1/2}}{{\pi }^{1/2}}+{Q}_{dl}+{Q}_{ads}$) and applying the slope of the resulting line (Q versus t^1/2^), the surface of the electrode was calculated as 0.065 cm^2^, which indicates an increase in the surface compared to its geometric surface area.

In the Anson equation, Q, n, A, F, C and D refers to the charge (coulombs), the transferred electron numbers, the electrochemical active surface area of the electrode (cm^2^), Faraday’s constant (96.485 coulombs/mole), the concentration and the diffusion coefficient (cm^2^/sec) of the probe respectively.

The use of bare and modified electrodes for measuring arsenite was investigated in this research and the results showed a further improvement in the performance of the electrode prepared with nanocomposite (Fig. S1b). In the observed voltammograms, the oxidation peak is related to the oxidation of arsenite, and the reduction peak is related to the reduction of arsenate to arsenite. The oxidation of arsenite to arsenate occurs at high potentials (above 1.5 V), which increases the possibility of interference with other species. Instead, the peak of arsenate reduction to arsenite appears at a suitable potential, which increases the significance of the measurement.

Considering that the oxidation of arsenite occurs at high potentials, in the case of using arsenite as the analyte, obtaining the reduction peak related to arsenate requires preliminary oxidation of the species by applying oxidative pretreatment, but if arsenate is used as the analyte, this pretreatment is not necessary. In scanning the potential in the reductive direction, the peak corresponding to the reduction of arsenate to arsenite appears at 0.5 V.

**Figure 5:**
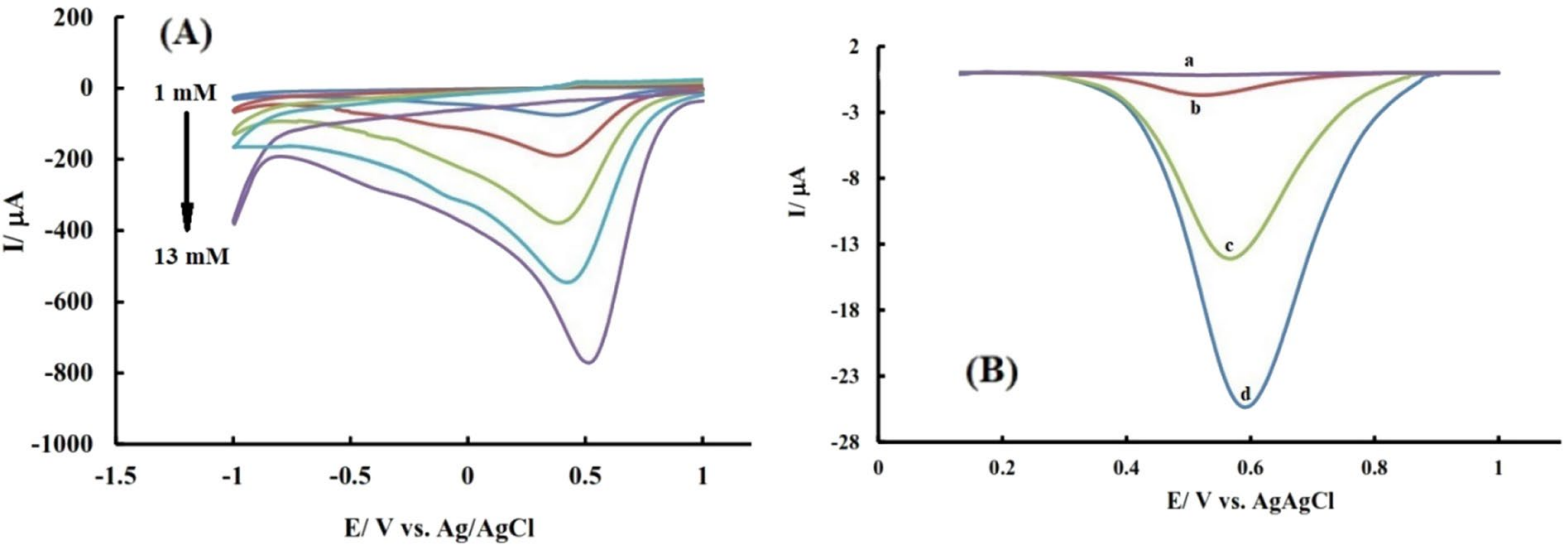
(**A**) CV results of nanocomposite modified electrode after 5 min dipping in AcBS containing different concentrations of arsenate (1, 3, 7, 10, and 13 mM); (**B**) DPV results of bare a: and modified electrodes b: AAGO-GCE, c: PDDA-PA/GCE, and d: AAGO-PDDA-PA/GCE after 5 min dipping in AcBS containing 15 μM As(V).

Cyclic voltammetry was used to investigate the effect of analyte concentration using solutions with different concentrations (Fig. 5A) and the results show a linear relationship between signal and concentration.

### Electrochemical measurement

Differential pulse voltammetry was used to measure low concentrations of arsenate and achieve low detection limits. In this regard, an example of differential pulse voltammograms obtained on the proposed modified electrode surface in the presence and absence of arsenate is shown in Fig. 5B.

As can be seen, with the addition of arsenate to the blank solution, the reduction signal appears and the observed increase at the surface of the nano-composite-modified electrode is greater than other electrodes. This result confirms the suitability of the proposed sensor for sensitive arsenate detection and is in good agreement with CV results.

**Figure 6:**
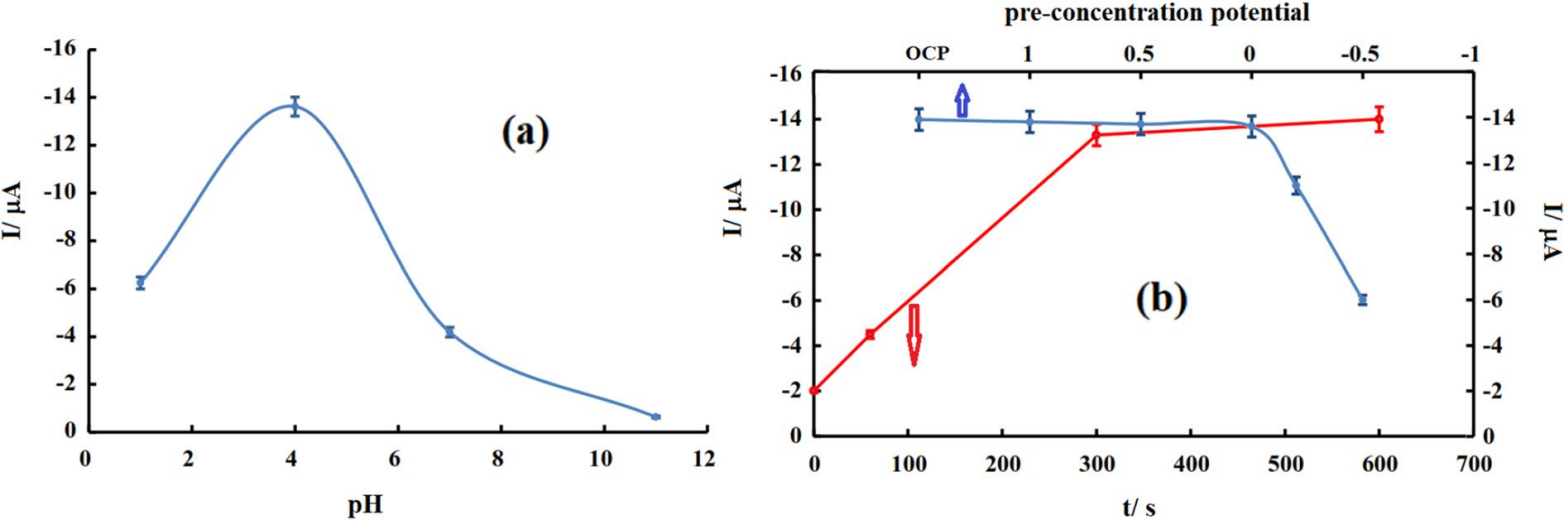
Variation of DPV response of nanocomposite modified electrode versus (**a**) pH and (**b**) accumulation time (red graph) and accumulation potential (blue graph). Accumulation conditions: AcBS containing 8 μM arsenate in OCP condition for different times or various potentials for 5 min.

Commonly, the acidity of the solution is very effective in the final results and this was also very evident in this research. Therefore differential pulse voltammetric behavior of nanocomposite-modified electrodes at different pH values was studied and results showed that the pH value is effective in measuring arsenic and the best peak height was obtained at a pH equal to 4 (Fig. 6a). Based on these results, it can be concluded that by lowering pH values, the arsenate species convert to arsenic acid and the adsorption of the uncharged species at the electrode surface is lower than charged one. Similarly, at alkaline pH, the adsorption of hydroxide and other possible anions on the surface prevents the adsorption of the analyte and the signal decreases. Moreover, at pH = 4, arsenite exists as a (natural) protonated species and cannot be adsorbed on the electrode surface, leading to higher arsenate adsorption and better sensor selectivity.

Considering the effect of pH on the results and the possibility of species adsorption on the electrode surface, pre-concentration time probably plays an important role in differential pulse voltammetry experiments, and with increasing time, the amount of analyte adsorbed on the electrode surface also increases. To investigate this factor and its effect on the arsenic detection process, the prepared electrodes were placed in a solution containing 5 μM arsenate with different pre-concentration times equal to 0, 1, 5, and 10 min and then their differential pulse voltammograms were recorded. This test was performed at open circuit potential for different time intervals (zero to 600 s). As can be seen (Fig. 6b), increasing the pre-concentration time by up to 5 min has a positive effect on the arsenate reduction signal on the electrode surface, and no change in the signal occurs at higher values. These results are related to the increase of adsorbed analyte with increasing time and saturation of the electrode surface in 5 min, so 5 min was chosen as the optimal preconcentration time. In addition, the effect of preconcentration time was studied in the cyclic voltammetry (data not shown) and the results are in good agreement with the DPV results.

### Investigating the pretreatment potential effect

Pretreatment potential is an important factor in obtaining reliable results in electrolysis processes.

Therefore, in this research, optimization of the pretreatment potential was done by two methods, cyclic voltammetry, and differential pulse voltammetry, and the related DPV results are presented in Fig. 6b. The experiment was carried out in 0.1 M acetate buffer solution with pH 4 containing arsenate applying different pre-concentration potentials for 5 min. As can be seen in the graphs, varying the potential in a positive direction has no effect on the resulting signal, and the signal obtained by performing open circuit preconcentration is equal to the resulting signal after applying 1 V. This result is probably related to the inherent positive charge of the electrode surface and its ability to adsorb arsenate. Applying negative potentials is not suitable for the accumulation of arsenate and the decrease in the obtained signal may be due to the electrostatic repulsion.

Investigations using arsenite as an analyte showed that it is necessary to apply a positive potential before recording the signal, which is probably related to the oxidation of the arsenite species to obtain the reduction signal. The results of differential pulse voltammetry are also consistent with the results of cyclic voltammetry.

### Concentration effect and detection limit

**Figure 7:**
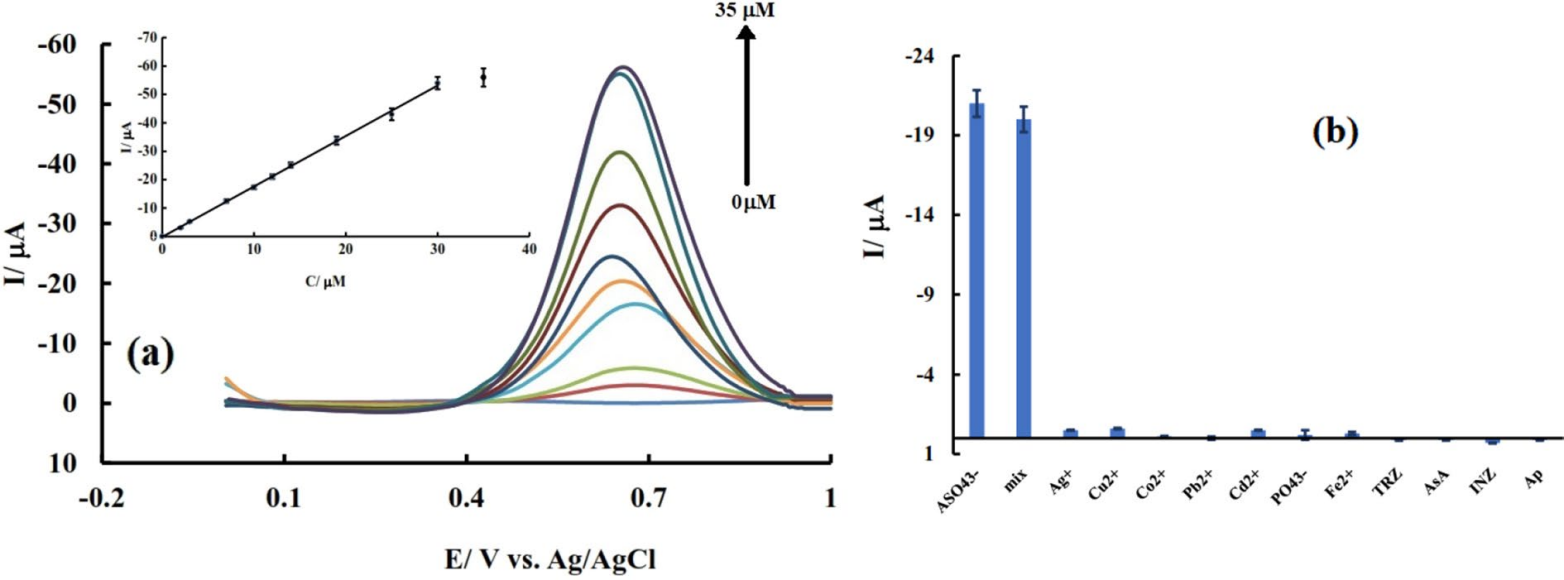
(**a**) DPV response of nanocomposite-modified GCE after preconcentration in OCP condition for 60 s in a stirred solution of As(V) with different concentrations (0, 2, 3, 7, 10, 12, 14, 19, 25, 30, 35 μM); (**b**) DPV response of nano-composite modified electrode after dipping in 12 μM AsO_4_^3–^ and possible interferents (Ag^+^, Cu^2+^, Co^2+^, Pb^2+^, Cd^2+^, PO_4_^3-^, Fe^2+^, tyrosine (TRZ), ascorbic acid (AsA), isoniazid (INZ), acetaminophen (AP) and their mixture plus AsO_4_^3^ (mix); Inset of a: Variation of current versus concentration of As(V).

The diagram of differential pulse voltammograms for the electrode modified with nano-composite in the presence of different concentrations of arsenate are shown in Fig. 7a. The linear dynamic range (LDR) of the sensor is up to 30 µM.

As can be seen in the graphs, with the increase of the concentration from 0 to 30 μM, the resulting signal also increased and the identification of the analyte was done in the best way. The graph of current changes versus concentration (calibration) shows the existence of a linear relationship based on equation $y\left(I/\mu A\right)=-1.79\left(C/\mu M\right)+0.23$ between signal and concentration and a correlation coefficient of 0.99 in this range (Fig. 7a-inset). The sensitivity of this sensor was 1.79 A/M. Considering the standard deviation of the sensor in the blank solution (S) and sensitivity or the slop of the calibration curve (m) based on equation the limit of detection (LOD) = 3S/m and the limit of quantification (LOQ) = 10S/m, LOD and LOQ of the sensor were calculated as 0.12µM and 0.38 μM. Comparing the results of this sensor with previous works shows the importance of the proposed selective sensor (Table 1).

**Table 1:** Comparison analytical parameters of AAGO-PDDA-PA/GCE with other modified electrodes as As sensor.

Modifier	Linear range	LOD	Ref
DWCNTs-Gr*	1 to 10 ppb	0.287 ppb	^24^
Fe_3_O_4_ -Au-IL*	1 to 100 µg/L	0.22 µg/L	^25^
AuNPs	–	16.73 µg L^−1^	^27^
NBBSH*	0.1 nM–0.1 M	50 pM	^49^
PAAP*	10.0 nM–0.1 M	6.8 nM	^50^
AuNPs-BDD*	0.1 to 1.5 ppm	20 ppb	^53^
AgNPs/PpyNW*	0.01 to 0.10 μM	1.5 ppb	^54^
carbon fiber ultra-microelectrodes/AuNPs	5 to 60 ppb	0.9 ppb	^55^
AgNPs/chit-GCE	10–100 ppb	1.20 ppb (16.2 nM)	^56^
Au-PANI-Fe-CNFs*	5–400 ppb	0.5 ppb	^57^
PtNPs-BDD*	Up to 100 ppb	0.5 ppb	^11^
AuNP-embedded carbon films	1–100 ppb	0.55 ppb	^58^
AAGO-PDDA-PA/GCE	Up to 30 μM	8.99 ppb	This work

*DWCNTs-Gr: double-walled carbon nanotubes and graphene hybrid thin film, Fe_3_O_4_-Au-IL: Magnetite-Decorated Gold Nanoparticles ionic liquide nanocomposite, NBBSH: (E)-N′ -(2-Nitrobenzylidene)-benzenesulfonohydrazide, PAAP: hybrid polyarylidene(azomethine-ether)s and copolyarylidene(azomethine-ether)s (PAAP) based on diarylidenecycloalkanones, AuNPs-BDD: gold nanoparticle-modified boron-doped diamond electrode, AgNPs/PpyNW: silver nanoparticles over single polypyrrole nanowire, Au-PANI-Fe-CNFs: AuNPs-polyaniline nanosheet array on on the electrospun Fe-containing carbon nanofibers, PtNPs-BDD: platinium nanoparticles-modified boron-doped diamond electrode.

### Selectivity and effect of interfering species

In order to investigate the selectivity of the proposed sensor, the voltammogram of a certain concentration of arsenate was recorded in the presence and absence of interfering factors and the resulting voltammetric currents were compared. If the signal change related to the analyte in the presence of interfering agents is less than 5%, it can be concluded that the investigated species does not interfere with the measurement of the analyte. The obtained results are summarized in Fig. 7b. Based on the investigations, most of the cations and some drugs mentioned in Fig. 7b, do not cause any disturbance in the measurement. Also, the results of Fig. 7b shows that the DPV response of the proposed sensor after interacting with arsenate is similar to the result of the sensor after interacting with a mixture of cations and arsenate. These results prove that there is no strong interaction between arsenate and the mentioned compounds. Therefore, the simultaneous presence of these species cannot interfere with the electrochemical detection of arsenate.

### Checking the stability of the electrode

The repeatability of the nanocomposite-modified GCE was checked by five times repeating the DPV test in 7 µM As(V) solution under optimal conditions, and the resulting RSD (3.31%) is a confirmation of the repeatability of the proposed sensor. Each prepared electrode could be used 9 times for detection purpose without significant change in results.

In order to check the reproducibility of the sensor preparation, the relative standard deviation was determined in the results obtained from five electrodes modified with nanocomposite, and the RSD equal to 3.91% indicates the good reproducibility of the proposed sensor.

One of the most important problems related to the presented sensor is its short time stability and the inability to reuse it on different days. In order to solve this problem, the possibility of using the pencil lead electrode (PLE) as an electrode substrate was also investigated, because due to PLE ’s low price and availability, it is possible to use it once. The obtained results showed that the signals obtained on the surface of the nanocomposite-modified GCE and the nanocomposite-modified PLE are not different and the optimal conditions obtained on the surface of the glassy carbon electrode can also be used in this case.

Finally, it can be said that the average response time, or in other words, the time required to reach the signal, is 6 min, including 5 min of accumulation and 1 min for recording the signal with stripping analysis, which all these characteristics indicate the suitability of the prepared electrode in sensor applications.

### Analysis of real sample

The nanocomposite-modified electrode was used for the determination of the inorganic arsenic amount in the tap water and rice flour samples. A standard addition method was applied to estimate the concentration of As in real environmental samples. For this purpose, after preparing the sample, a certain volume (10 ml) of the sample solution was poured into the electrochemical cell, and the corresponding voltammogram was recorded under optimal conditions. Then, increasing volumes of the standard solutions of the 1 M analyte (20, 60, 100, 150, 300 µL) were added to this solution. At each increment of the analyte, the voltammetric response is recorded and the data are measured. The calibration curve is drawn according to the standard concentration, and through extrapolation and obtaining the x-intercept, the concentration of the unknown sample is obtained. The results showed that in the studied samples, the concentration of arsenic ions is lower than the detection limit of the proposed sensor. Also, considering the amount of dilution, it is clear that the amount of arsenic is lower than the allowed amount determined according to the national standard of Iran (0.15 ppm).

To verify the accuracy (validaty) of the results, different amounts of As(V) standard solution were added to the sample matrix, and after measuring the amount of arsenic, the recovery percentage was calculated. Based on the calculated recovery (Table S1) and RSD % obtained, this method can be used to determine arsenic in rice and water samples.

## Conclusions

A multistep electrode preparation method composed of PDDA, PA, crosslinker, and AAGO deposition was used for the determination of As(V) as arsenate. The combination of these modifiers at the electrode surface led to the production of a new sensor for the selective determination of As(V) in real samples. The presence of positively charged PDDA, and conductive PA in addition to nanometric AAGO resulted in good adsorption of negatively charged analyte AsO_4_^3-^, and higher voltammetric signals. The study of interference of the tested elements proved selective determination of As(V). Finally, this sensor was successfully applied for measuring As(V) in rice and water samples. Low cost, ease of use, biocompatibility, high selectivity, sensitivity, and reproducibility are the advantages of the proposed sensor. Due to the limited stability of the proposed sensor (only 1 day), the possibility of using a pencil electrode was investigated and similar results were obtained. In order to save time, in future works, the possibility of using screen-printed graphite electrodes will be investigated, which is being done in our laboratory.

## Supplementary Information

Supplementary Information.

## Data Availability

All data generated or analysed during this study are included in this published article and its supplementary information files.

## References

[1] Cui L, Wu J, Ju H (2016). Label-free signal-on aptasensor for sensitive electrochemical detection of arsenite. Biosens. Bioelectron..

[2] http://www.who.int/int-fs/en/fact210.html (1993).

[3] Jia X, Gong D, Wang J, Huang F, Duan T, Zhang X (2016). Arsenic speciation in environmental waters by a new specific phosphine modified polymer microsphere preconcentration and HPLC–ICPMS determination. Talanta.

[4] Álvarez-Llamas G, del Rosario Fernández de laCampa MA, Sanz-Medel A (2005). ICP-MS for specific detection in capillary electrophoresis. TrAC Trends Anal. Chem..

[5] Feng Y, Chen H, Tian L, Narasaki H (1998). Off-line separation and determination of inorganic arsenic species in natural water by high resolution inductively coupled plasma mass spectrometry with hydride generation combined with reaction of arsenic (V) and L-cysteine. Anal. Chim. Acta.

[6] Macedo SM, de Jesus RM, Garcia KS, Hatje V, de Queiroz AFS, Ferreira SLC (2009). Determination of total arsenic and arsenic (III) in phosphate fertilizers and phosphate rocks by HG-AAS after multivariate optimization based on Box-Behnken design. Talanta.

[7] Zhang N, Fu N, Fang Z, Feng Y, Ke L (2011). Simultaneous multi-channel hydride generation atomic fluorescence spectrometry determination of arsenic, bismuth, tellurium and selenium in tea leaves. Food Chem..

[8] Hung DQ, Nekrassova O, Compton RG (2004). Analytical methods for inorganic arsenic in water: A review. Talanta.

[9] Dai X, Compton RG (2005). Gold nanoparticle modified electrodes show a reduced interference by Cu(II) in the detection of As(III) using anodic stripping voltammetry. Electroanalysis.

[10] Cavicchioli A, La-Scalea MA, Gutz IGR (2004). Analysis and speciation of traces of arsenic in environmental, food and industrial samples by voltammetry: A review. Electroanalysis.

[11] Hrapovic S, Liu Y, Luong JHT (2007). Reusable platinum nanoparticle modified boron doped diamond microelectrodes for oxidative determination of arsenite. Anal. Chem..

[12] Dai X, Compton RG (2006). Direct electrodeposition of gold nanoparticles onto indmium tin oxide film coated glass: Application to the detection of arsenic (III). Anal. Sci..

[13] Dreyer DR, Park S, Bielawski CW, Ruoff RS (2010). The chemistry of graphene oxide. Chem. Soc. Rev..

[14] Fan Y, Han C, Zhang B (2016). Recent advances in the development and application of nanoelectrodes. Analyst.

[15] Alam MM, Rashed MA, Rahman MM, Rahman MM, Nagao Y, Hasnat MA (2018). Electrochemical oxidation of As (III) on Pd immobilized Pt surface: Kinetics and sensing performance. RSC Adv..

[16] Ghosh SK, Mandal M, Kundu S, Nath S, Pal T (2004). Bimetallic Pt–Ni nanoparticles can catalyze reduction of aromatic nitro compounds by sodium borohydride in aqueous solution. Appl. Catal. A.

[17] Oja SM, Wood M, Zhang B (2013). Nanoscale electrochemistry. Anal. Chem..

[18] Gupta R, Gamare JS, Pandey AK, Tyagi D, Kamat JV (2016). Highly sensitive detection of arsenite based on its affinity toward ruthenium nanoparticles decorated on glassy carbon electrode. Anal. Chem..

[19] Profumo A, Fagnoni M, Merli D, Quartarone E, Protti S, Dondi D, Albini A (2006). Multiwalled carbon nanotube chemically modified gold electrode for inorganic As speciation and Bi(III) determination. Anal. Chem..

[20] Ramesha GK, Sampath S (2011). In-situ formation of graphene–lead oxide composite and its use in trace arsenic detection. Sens. Actuators B Chem..

[21] Guardia P (2016). Electrocatalytic nanocauliflower structured fluorine doped CdO thin film as a potential arsenic sensor. Sens. Actuators B Chem..

[22] Yang M, Guo Z, Li L-N, Huang Y-Y, Liu J-H, Zhou Q, Chen X, Huang X-J (2016). Electrochemical determination of arsenic(III) with ultra-high anti-interference performance using Au–Cu bimetallic nanoparticles. Sens. Actuators B Chem..

[23] Gibbon-Walsh K, Salaün P, van den Berg CMG (2012). Determination of arsenate in natural gold pH seawater using a manganese-coated microwire electrode. Anal. Chim. Acta.

[24] Duoc PND (2020). A novel electrochemical sensor based on double-walled carbon nanotubes and graphene hybrid thin film for arsenic(V) detection. J. Hazard Mat..

[25] Sedki M, Zhao G, Ma S, Jassby D, Mulchandani A (2021). Linker-free magnetite-decorated gold nanoparticles (Fe_3_O_4_-Au): Synthesis, characterization, and application for electrochemical detection of arsenic (III). Sensors.

[26] Salunke RS, Nakate YT, Umar A, Nakate UT, Ahmad R, Shirale DJ (2021). Anodic stripping voltammetry analysis of gold nanoparticles functionalized one-dimensional single polypyrrole nanowire for arsenic sensing. Surf. Interfaces.

[27] Sullivan C, Lu D, Senecal A, Kurup P (2021). Voltammetric detection of arsenic (III) using gold nanoparticles modified carbon screen printed electrodes: Application for facile and rapid analysis in commercial apple juice. Food Chem..

[28] Zong C, Jin X, Liu J (2021). Critical review of bio/nano sensors for arsenic detection. Trends Environ. Anal. Chem..

[29] Dautzenberg HE, Görnitz E, Jaeger W (1998). Synthesis and characterization of pol (diallyldimethylammonium chloride) in a broad range of molecular weight. Macromol. Chem. Phys..

[30] Jia L, Liu J, Wang H (2013). Preparation of poly(diallyldimethylammonium chloride)-functionalized graphene and its applications for H_2_O_2_ and glucose sensing. Electrochim. Acta.

[31] Li X, Zhong A, Wei S, Luo X, Liang Y, Zhu Q (2015). Polyelectrolyte functionalized gold nanoparticles-reduced graphene oxide nanohybrid for electrochemical determination of aminophenol isomers. Electrochim. Acta.

[32] Ensafi A, Gorgabi-Khorzoughi M, Rezaei B, Jafari-Asl M (2017). Electrochemical behavior of polyoxometalates decorated on poly diallyl dimethyl ammonium chloride-MWCNTs: A highly selective electrochemical sensor for determination of guanine and adenine. J. Taiwan Inst. Chem. Eng..

[33] Gao F, Yang J, Tu X, Yu Y, Liu S, Li M, Gao Y, Wang X, Lu L (2021). Facile synthesis of ZIF-8@poly(3,4-ethylenedioxythiophene): poly (4-styrenesulfonate) and its application as efficient electrochemical sensor for the determination dichlorophenol. Synth. Met..

[34] Xu G (2013). Electrodeposited conducting polymer PEDOT doped with pure carbon nanotubes for the detection of dopamine in the presence of ascorbic acid. Sens. Actuators B Chem..

[35] Namsheer K, Rout CS (2021). Conducting polymers: a comprehensive review on recent advances in synthesis, properties and applications. RSC Adv..

[36] Naveen MH, Gurudatt NG, Shim Y-B (2017). Applications of conducting polymer composites to electrochemical sensors: A review. Appl. Mater. Today.

[37] Terán-Alcocer Á (2021). Electrochemical sensors based on conducting polymers for the aqueous detection of biologically relevant molecules. Nanomaterials.

[38] Martin CR (1995). Template synthesis of electronically conductive polymer nanostructures. Acc. Chem. Res..

[39] Dinh LNM, Tran BN, Agarwal V, Zetterlund PB (2022). Synthesis of highly stretchable and electrically conductive multiwalled carbon nanotube/polymer nanocomposite films. ACS Appl. Polym. Mater..

[40] Rahman MM, Alenazi NA, Hussein MA, Alam MM, Alamry KA, Asiri AM (2018). Nanocomposites-based nitrated polyethersulfone and doped ZnYNiO for selective As^3+^ sensor application. Adv. Polym. Technol..

[41] Manjunatha JG (2020). Poly (adenine) modified graphene-based voltammetric sensor for the electrochemical determination of catechol, hydroquinone and resorcinol. Open Chem. Eng. J..

[42] Hareesha N, Manjunatha JG, Amrutha BM, Sreeharsha N, Basheeruddin SM, Anwer AMdK (2021). A fast and selective electrochemical detection of vanillin in food samples on the surface of poly(glutamic acid) functionalized multiwalled carbon nanotubes and graphite composite paste sensor. Colloids Surf. A.

[43] Hareesha N, Manjunatha JG, Amrutha BM, Pushpanjali PA, Charithra MM, Prinith Subbaiah N (2021). Electrochemical analysis of indigo carmine in food and water samples using a poly (glutamic acid) layered multi-walled carbon nanotube paste electrode. J. Electron. Mater..

[44] Manjunatha JG (2019). Electrochemical polymerised graphene paste electrode and application to catechol sensing. Open Chem. Eng. J..

[45] Monnappa AB, Manjunatha JGG, Bhatt AS, Nagarajappa H (2021). Sensitive and selective electrochemical detection of vanillin at graphene based poly (methyl orange) modified electrode. J. Sci..

[46] Manjunatha JG (2016). Poly (nigrosine) modified electrochemical sensor for the determination of dopamine and uric acid: A cyclic voltammetric study. Int. J. Chem. Tech. Res..

[47] Manjunatha JG, Deraman M, Basri NH (2015). Electrocatalytic detection of dopamine and uric acid at poly (basic blue B) modified carbon nanotube paste electrode. Asian J. Pharm. Clin. Res..

[48] Manjunatha J (2012). Selective determination of dopamine in the presence of ascorbic acid using a poly (Nicotinic Acid) modified carbon paste electrode. Anal. Bioanal. Electrochem..

[49] Rahman MM, Hussain MM, Arshad MN, Awual MdR, Asiri AM (2019). Arsenic sensor development based on modification with (E)-N′-(2-nitrobenzylidine)-benzenesulfonohydrazide: A real sample analysis. New J. Chem..

[50] Rahman MM, Hussein MA, Aly KI, Asiri AM (2018). Thermally stable hybrid polyarylidene (azomethine-ether) s polymers (PAAP): An ultrasensitive arsenic (III) sensor approach. Des. Monomers Polym..

[51] Awual MdR, Hasan MdM, Asiri AM, Rahman MM (2019). Cleaning the arsenic(V) contaminated water for safe-guarding the public health using novel composite material. Compos. B Eng..

[52] Ghasemi Kochameshki M, Marjani A, Mahmoudian M, Farhadi K (2017). Grafting of diallyldimethylammonium chloride on graphene oxide by RAFT polymerization for modification of nanocomposite polysulfone membranes using in water treatment. Chem. Eng. J..

[53] Pungjunun K, Chaiyo S, Jantrahong I, Nantaphol S, Siangproh W, Chailapakul O (2018). Anodic stripping voltammetric determination of total arsenic using a gold nanoparticle-modified boron-doped diamond electrode on a paper-based device. Microchim. Acta.

[54] Salunke RS, Chavan PG, Shirale DJ (2018). Anodic stripping voltammetry studies of electrochemically engineered silver nanoparticles over single polypyrrole nanowire device for tracing of arsenic(III): An environmental perspective. Nanotechnol. Environ. Eng..

[55] Fernández L, Carrera P, Romero H, Alvarado J, Espinoza-Montero PJ (2017). Electrochemical determination of arsenic in natural waters using carbon fiber ultra-microelectrodes modified with gold nanoparticles. Talanta.

[56] Prakash S, Chakrabarty T, Singh AK, Shahi VK (2012). Silver nanoparticles built-in chitosan modified glassy carbon electrode for anodic stripping analysis of As(III) and its removal from water. Electrochim. Acta.

[57] Tang Q, Zhu G, Ge Y, Yang J, Huang M, Liu J (2020). AuNPs-polyaniline nanosheet array on carbon nanofiber for the determination of As (III). J. Electroanal. Chem..

[58] Kato D, Kamata T, Kato D, Yanagisawa H, Niwa O (2016). Au nanoparticle-embedded carbon films for electrochemical As^3+^ detection with high sensitivity and stability. Anal. Chem..

