# Supplementary material for: An electrochemical sensor for the detection of arsenic using nanocomposite-modified electrode
Source: Sci Rep. 2023 May 31;13:8816. doi: 10.1038/s41598-023-36103-6 (PMC10232514; doi:10.1038/s41598-023-36103-6)
Supplement: Supplementary file 1 — Supplementary Information. [file 41598_2023_36103_MOESM1_ESM.docx]

**An electrochemical sensor for detection of arsenic using nano composite modified electrode**

S. Hamid Kargari^a^, F. Ahour,^a,b*^ and M. Mahmoudian^a,b^

*^a^Nanotechnology Research Group, Faculty of Science, Urmia University, Urmia, Iran*

*^b^Department of Nanochemistry, Nanotechnology Research Center, Urmia University, Urmia, Iran*;

Corresponding author:

* Fatemeh Ahour

E-mail: [Fatemeh.ahour@gmail.com](mailto:Fatemeh.ahour@gmail.com), f.ahour@urmia.ac.ir; Fax: +98 44-32752746

**RESULTS AND DISCUSSION**

**Preliminary experiments**

Figure S1. CV experiments of bare and modified electrodes in (a) 5 mM ferri-ferro containing 0.5 M NaCl; (b) in 10 mM arsenite solution. scan rate: 50 mV s^-1^.

**Concentration effect and detection limit**

Table S1. Measurement results of As in rice powder and water samples

| Sample | AsO_4_^3-^added (μM) | AsO_4_^3-^ found (nM) | Recovery (%) | RSD (%) [a] |
| --- | --- | --- | --- | --- |
| rice powder | 0 | 0 |  | 1.9 |
|  | 2 | 2.09 | 104 | 2.8 |
|  | 5 | 4.93 | 98.6 | 3.3 |
| Tap water | 0  3  6 | 0  3.11  6.19 | 103.6  103.2 | 1.8  2.1  2.6 |
